# Efficacy and Safety of Vedolizumab in Patients with Inflammatory Bowel Disease in Association with Vedolizumab Drug Levels

**DOI:** 10.3390/jcm13010140

**Published:** 2023-12-27

**Authors:** Eva Hüttemann, Anna Muzalyova, Katharina Gröhl, Sandra Nagl, Carola Fleischmann, Alanna Ebigbo, Johanna Classen, Julia Wanzl, Friederike Prinz, Patrick Mayr, Elisabeth Schnoy

**Affiliations:** 1 Internal Medicine III, University Hospital Augsburg, 86156 Augsburg, Germany; anna.muzalyova@uk-augsburg.de (A.M.); katharina.groehl@uk-augsburg.de (K.G.); sandra.nagl@uk-augsburg.de (S.N.); carola.fleischmann@klinikum-nuernberg.de (C.F.); alanna.ebigbo@uk-augsburg.de (A.E.); johanna-maria.classen@uk-augsburg.de (J.C.); julia.wanzl@uk-augsburg.de (J.W.); rik.pri@gmx.de (F.P.); 2 Internal Medicine, Kantonsspital St. Gallen, 9007 St. Gallen, Switzerland; 3 Department of Gastroenterology, Hepatology and Endocrinology, Klinikum Nürnberg, 90419 Nuremberg, Germany; 4 Internal Medicine II, University Hospital Augsburg, 86156 Augsburg, Germany; patrick.mayr@kssg.ch; 5 Department of Oncology and Hematology, Kantonsspital St. Gallen, 9007 St. Gallen, Switzerland

**Keywords:** inflammatory bowel disease, vedolizumab, drug level, ulcerative colitis, Crohn’s disease

## Abstract

Background: Vedolizumab (VDZ) is a well-established and important therapeutic option in the treatment of patients with inflammatory bowel disease (IBD). However, the significance of therapeutic drug monitoring (TDM) with VDZ remains a contradictory field in daily clinical practice. Our study aims to clarify the predictive impact of VDZ drug levels in long-term clinical outcomes in a real-world cohort. Methods: Patients with moderate to severe ulcerative colitis (UC) and Crohn’s disease (CD) from a tertiary IBD referral center at the University Hospital Augsburg, Germany, were enrolled in this single-center retrospective data analysis. Clinical and endoscopic data were collected at month 6, month 12, and at the last time of follow-up, and outcomes were correlated with VDZ levels at week 6. Results: This study included 95 patients, 68.4% (*n* = 65) with UC, 24.2% (*n* = 23) with CD, and 7.4% (*n* = 7) with indeterminate colitis (CI). Patients with a mean VDZ treatment time of 17.83 months ± 14.56 showed clinical response in 29.5% (*n* = 28) and clinical remission in 45.3% (*n* = 43) at the end of the study. Endoscopic response occurred in 20.0% (*n* = 19) and endoscopic remission in 29.5% (*n* = 28) at the end of the study. The sustained beneficial effect of VDZ was also reflected in a significant change in biomarker levels. VDZ trough level at week 6 was determined in 48.4% (*n* = 46) with a mean of 41.79 µg/mL ± 24.58. A significant association between VDZ level at week 6 and both short and long-term outcomes could not be demonstrated. However, numerically higher VDZ levels were seen in patients with endoscopic and clinical improvement at month 6 and at the time of last follow-up. Conclusions: This study demonstrated efficacy and safety for VDZ in a real-world cohort. Although, for some parameters, a clear trend for higher VDZ levels at week 6 was seen, the efficacy of VDZ was not significantly correlated to VDZ level at week 6, which questions the predictive value of VDZ levels in the real world.

## 1. Introduction

Both Crohn’s disease (CD) and ulcerative colitis (UC) belong to the inflammatory bowel diseases (IBD), a disease with an increasing incidence every year [1]. Clinical symptoms can vary from asymptomatic to severe and life-threatening, including (bloody) diarrhea, weight loss, and abdominal pain. While CD is known to affect the gastrointestinal (GI) tract from oral to anal, mostly segmentally, and is more frequently associated with local complications, UC mainly involves the distal or complete colon [2,3]. In addition to extraintestinal manifestations that can occur, the risk of developing colorectal cancer (CRC) is also slightly increased [4]. The exact underlying mechanism in the disease development of IBD is unclear, but a multifactorial disease cause is postulated, including genetic, microbial, and environmental factors [5]. Besides clinical remission, the primary aim is to achieve “mucosal healing (MH)” [6]. In addition to conventional therapies including aminosalicylates, corticosteroids, and immunosuppressants, various targeted biologics have increasingly entered clinical routine over the past years [7].

One of these targeted therapies is vedolizumab (VDZ), a monoclonal antibody and α4β7 integrin inhibitor approved in 2014 by the European Medicines Agency (EMA) in Europe [8]. By interfering with the interaction between α4β7 and the mucosal cell adhesion molecule adhesion molecule-1, it selectively prevents the transport of leukocytes to the intestinal wall [8]. Although VDZ has significantly improved long-term remission in clinical practice, not every patient benefits from this therapy. Data have shown that the incidence rates for loss of response were 47.9/100.000 patient years for CD and 39.8/100.000 patient years for UC [9].

One possible approach to optimize and improve therapy with VDZ in IBD is therapeutic drug monitoring (TDM). TDM is already well established for TNF inhibitors. Due to the dose-dependent therapeutic effect of TNF inhibitors, long-term remission may be achieved by dose escalation, shorter infusion intervals, or combination therapy, controlled by measuring drug levels and antibodies [10]. However, data for VDZ and drug monitoring remain inconclusive.

The GEMINI trials revealed that higher VDZ drug levels at week 6 correlate with an increased clinical response rate [11,12]. Furthermore, there are signs that dose intensification may rescue more than half of patients with a loss of effect on VDZ over time [9,10].

Due to the scarce data available so far, the aim of this study was to focus on the treatment response of VDZ in patients with IBD in a real-world cohort in a single center and correlate the results to the drug levels at week 6 with clinical data in the long-term.

## 2. Material and Methods

This study was a single-center retrospective data analysis at a tertiary IBD referral center at the University Hospital Augsburg in Augsburg, Germany. The study included patients receiving VDZ between March 2014 and January 2022. The last follow-up was the time of the last VDZ administration or last contact until January 2022. Adult patients 18 years and older and with a confirmed diagnosis of moderate to severe CD or UC and a complete induction therapy of at least 4 infusions of VDZ were included. Exclusion criteria were contraindications to VDZ as described in the prescribing information. All the data were retrospectively recorded from the available electronic medical charts.

Besides the ineffectiveness of prior therapy, the synopsis of clinical status, biomarkers, imaging, and endoscopy was essential for the decision to treat with VDZ. For induction, all patients received 300 mg VDZ intravenously (i.v.) at weeks 0, 2, and 6, after that, every 8 weeks intravenously or subcutaneously (s.c.). In case of insufficient response at week 6 an additional 300 mg infusion of VDZ was given at week 10. Primary nonresponders or patients with a loss of response received a therapy escalation, which means shorter infusion intervals of VDZ (every 4 or 6 weeks).

A second induction with VDZ after an initial failure to respond to primary treatment was called re-induction.

Clinical and endoscopic activity, as well as blood samples, were retrieved at baseline, week 6, 6 months, and 12 months and at the time of last follow-up. Clinical response was defined as a substantial improvement in disease symptoms, and clinical remission was defined as the complete absence of symptoms. To assess the clinical outcomes, the treating physician took into account the patient’s symptom burden, biomarkers, and imaging; no score was used. Endoscopic response and remission were verified through endoscopy findings during follow-up and were supported by histopathologic findings when appropriate.

The following data were collected: age, sex, IBD type and duration, age at diagnosis, body mass index (BMI), extraintestinal manifestations, disease severity, previous IBD therapies, mode of VDZ application, need for an additional infusion at week 10 and/or treatment escalation, clinical and endoscopic activity, concomitant use of steroids, and adverse events (AE).

For data analysis, we used the following serum parameters taken before VDZ infusion: C-reactive protein (CRP, reference range [rr] 0–0.5 mg/dL), hemoglobin (Hb, rr 140–180 g/L), ferritin as a marker of iron status (Fe, rr 30–400 ng/mL) and albumin (Alb, rr 35–52 g/L). Calprotectin (Clp, rr < 50 µg/g) was recorded within one year after baseline measurement, and the VDZ serum concentration (µg/mL) was determined at week 6. Our certified central hospital laboratory analyzed the collected serum samples except for VDZ serum level, which was tested by the external laboratory Limbach in Heidelberg, Germany, using a tryptic digestion and a liquid chromatography–mass spectrometry (Xevo TQ-XS Triple Quadrupol mass spectrometer, Waters, Eschborn, Germany).

This study was performed in accordance with Good Clinical Practice and with the Declaration of Helsinki. It was approved by the Ethics Committee at the University of Regensburg, Regensburg, Germany (Nr. 23-3212-104).

Based on real-world data, the primary aim was to investigate the correlation of VDZ level at week 6 after induction therapy with VDZ in patients with CU and CD with the long-term outcome and whether it is a possible prognostic factor for remission induction.

The secondary outcomes were:

- Characterization of the cohort receiving VDZ;
- Impact of the number of prior therapies on treatment response;
- Short- and long-term outcomes between patients with and without VDZ level at week 6;
- Adverse events.

Patient demographics and baseline characteristics were stated using descriptive statistics. Continuous variables are presented as mean values and standard deviations as minimum, maximum, and range. Categorical variables are reported in absolute numbers and percentages. A comparison of the means of two independent groups was conducted using the Mann–Whitney *U* test. The Wilcoxon signed-rank test was used to analyze paired samples. The Kruskal–Wallis test was performed to determine differences between more than two independent samples followed by pairwise comparison if significant. To investigate relationships between continuous variables, the Spearman correlation was used. Fisher’s exact test or chi-squared test were used to assess the association between categorical variables. The significance level was set at α = 0.05. Data management, descriptive, and interference-statistical analysis were conducted using IBM SPSS Version 27. Graphics were performed using Excel Version 2303. Re-inductions were not included in the statistics but were treated separately.

## 3. Results

### 3.1. Patients Demographics

Our study included 95 patients with an equal sex distribution (females *n* = 47 (49.5%), males *n* = 48 (50.5%) (Table S1). A total of 65 patients (68.4%) with UC and 23 patients (24.2%) with CD were enrolled. Seven (7.4%) patients could not be assigned to one diagnosis and formed the colitis indeterminata (CI) group. The mean age of the study collective at diagnosis was 30.87 years ± 14.68, and at the start of VDZ therapy, 41 years ± 15.28.

### 3.2. Disease Characteristics at Baseline

At baseline, 67 patients (70.5%) (Table S2) reported a moderate to high symptom burden such as (bloody) diarrhea or weight loss. Only five patients (5.3%) were in clinical remission. In 23 patients (24.2%), the exact clinical activity was not available. The cohort had a mean BMI of 23.6 kg/m^2^ ± 4.41 before VDZ was applied.

Overall, 32 patients (33.7%) showed at least one extraintestinal manifestation of which nine patients (28.1%) had two extraintestinal manifestations. The most common extraintestinal manifestations were joint involvements such as arthralgia (*n* = 20, 48.8%) (Table S3), followed by eye involvements (uveitis, iritis, or episcleritis) (*n* = 8, 19.5%) and the presence of primary sclerosis cholangitis (PSC) (*n* = 6, 14.6%).

Forty-three patients (45.3%) had experienced one or more IBD-associated complications before treatment with VDZ. The most frequent complications included stenosis (*n* = 23, 30.3%), fistulas (*n* = 16, 21.1%), and abscesses (*n* = 11, 14.5%). All IBD-associated complications are listed in Table 1.

Twenty-five patients (26.3%) of the population had bowel surgery before VDZ treatment, most frequently due to local complications such as abscess splitting and fistula repair, and less frequently due to uncontrollable inflammation or malignancies. Of these, eight were UC patients (32.0%) and 17 suffered from CD (68.0%). Comparing patients with CD and UC, there was a significantly longer duration of disease in CD than in UC at the time of last follow-up (15.53 years ± 7.04 vs. 11.48 years ± 9.30, *p* ≤ 0.01) (Table S4).

Baseline biomarkers are shown in Table 2 and Table S5. In comparison between CD and UC, patients with CD presented with significantly higher Fe levels at baseline (274.74 ng/mL ± 399.62 vs. 108.02 ng/mL ± 166.03, *p* = 0.036). No differences were seen in Alb levels (41.83 g/L ± 5.94 vs. 42.19 g/L ± 5.25, *p* = 0.812), in Clp levels (495.05 µg/g ± 293.13 vs. 473.5 µg/g ± 328.16, *p* = 0.618), in CRP levels (1.77 mg/dL ± 1.96 vs. 1.56 mg/dL ± 2.99, *p* = 0.177), or in Hb levels (132.91 g/L ± 13.77 vs. 126.67 g/L ± 19.40, *p* = 0.524).

Before therapy initiation, 79 (83.2%) endoscopies were documented, of which 77 (97.5%) reported inflammatory activity, and two endoscopies (2.5%) showed endoscopic remission. Sixteen records (16.8%) were not available.

Thirty-three patients (34.7%) underwent bowel ultrasound evaluation at baseline. Of these 33 ultrasounds, 23 (69.7%) were pathological and showed wall thickening or separation, hyperperfusion, stenosis, or abnormal lymph nodes. Ten findings (30.3%) were physiological. 

All 18 cross-sectional imaging examinations (19.0%) performed before therapy initiation showed pathologic findings such as inflammatory activity (*n* = 16), stenosis (*n* = 6), suspicious lymph nodes (*n* = 3), wall thickening (*n* = 2), fistulas (*n* = 2), or hyperperfusion (*n* = 1).

### 3.3. Prior Therapies

Patients in the study had an average of 2.7 ± 1.70 prior therapies before VDZ therapy. Fifty-one patients (54.3%) had zero to two previous treatments. Thirty-one patients (33.0%) had three or four prior therapies, and twelve (12.8%) received five or more therapies before starting VDZ. Of all the patients’ prior therapy lines combined, a total of 254 therapy lines were administered over time. Of these 254 therapy lines, therapy regimes were monotherapy in 87.4% (*n* = 222), dual combination therapy in 11.8% (*n* = 30), and triple combination therapy in 0.8% (*n* = 2), as shown in Table 3. Table 3 also lists the frequency of the various used agents before VDZ.

A strong, significant positive correlation was found between the number of prior therapies and the disease duration (rho = 0.569, *p* ≤ 0.01) (Table S6). On the other hand, the remission duration showed a moderate negative correlation with the number of prior therapies (rho −0.289, *p* ≤ 0.01). The number of previous treatments was also significantly higher in patients with CD than in UC (4.0 ± 1.57 vs. 2.26 ± 1.52, *p* ≤ 0.01) (Table S7).

### 3.4. Characteristics of VDZ Treatment

Patients in this study had an average disease duration of 10.5 years ± 9.0 (Table S1) before starting VDZ. VDZ therapy duration lasted for an average of 17.8 months ± 14.6 at the time of the last follow-up. Some patients continued VDZ therapy afterward, as described below. Eighty-two patients (86.3%) received VDZ i.v. exclusively, and thirteen patients (13.7%) switched to VDZ s.c. An additional administration of VDZ at week 10 was given to 35 patients (36.8%). There was no significant difference in additive administration of VDZ at week 10 between patients with CD (*n* = 12) or UC (*n* = 22, *p* = 0.217). Therapy escalation (i.e., shortening of the eight-week interval) was required in 62 patients (65.3%) due to either loss of response or insufficient response. Re-induction (i.e., second induction with VDZ after initial failure to respond to primary treatment) was performed in two patients (2.1%), presented in the supplementary material. At the time of the last follow-up, 47 patients (49.5%) had maintained VDZ and 48 (50.5%) discontinued therapy with VDZ. The most common reason for treatment discontinuation was loss of response during the course (secondary nonresponder, *n* = 21, 43.8%). In 19 cases (39.6%), therapy was discontinued due to insufficient response (primary nonresponder). Eight patients (16.7%) discontinued treatment as their own decision (e.g., desire to have children, moving away, incompliance).

### 3.5. Clinical Outcome

Six months after therapy initiation, 24.2% (*n* = 23) (Table S8) showed a clinical response, 13.7% (*n* = 13) were in remission, and 16.8% (*n* = 16) were refractory. A total of 45.3% (*n* = 43) of the information was unavailable due to VDZ therapy duration of less than six months, external therapy initiation, or a lack of documentation. Twelve months after beginning with VDZ, 8.4% (*n* = 8) showed a clinical response, 13.7% (*n* = 13) showed remission, and 9.5% (*n* = 9) were refractory. A total of 68.4% of the information was not available. Looking at the clinical course of individual patients between month 6 and 12, out of the twemty-three patients who initially responded at month 6, three patients (13.0%) continued to show clinical response at month 12, four patients (17.4%) achieved remission, and ten (43.5%) either showed loss of response or had discontinued therapy. In six cases (26.1%), documentation was not available.

Out of the thirteen patients with clinical remission at month 6, five patients (38.5%) still showed remission at 12 months and one (7.7%) showed a clinical response. Four patients discontinued therapy (30.8%), and three cases (23.1%) remained unknown. Of the sixteen patients who were refractory at month 6, only one patient (6.3%) achieved clinical response at month 12. Twelve patients (75.0%) either remained refractory at month 12 or had already discontinued therapy. In three cases (18.8%), documentation was not available.

Overall, at the time of the last follow-up, 29.5% (*n* = 28) showed clinical response, 45.3% (*n* = 43) patients were in remission, 16.8% (*n* =16) were refractory, 3.2% (*n* = 3) did not improve or worsen with VDZ and were considered as stable, and five cases (5.3%) were not documented.

There was a tendency for higher treatment response in men (women 20.5% vs. men 41.3%) and better remission induction in women (women 61.4% vs. men 37.0%); however, the difference was not significant (*p* = 0.098) (Table S9). There was also no significant difference in clinical outcome between patients with CD and UC (*p* = 0.331) (Table S10) and in patients who received therapy escalation (*p* = 0.159) (Table S11).

Regarding the clinical outcome and the number of prior therapies, 3.9% of patients (*n* = 2) with 0–2 previous treatments were clinically refractory. A total of 29.0% of patients (*n* = 9) with three to four prior therapies and 41.7% (*n* = 5) with five or more prior treatments were refractory. Therefore, the more refractory the clinical outcome, the more prior therapies (*p* = 0.022) a patient had received. Clinical response and remission decreased numerically with an increased number of previous treatments.

### 3.6. Endoscopic Outcome

Six months after the initiation of therapy with VDZ, 15.8% of patients (*n* = 15) (Table S12) showed an endoscopic response, 12.6% (*n* = 12) were endoscopically in remission, and 17.9% (*n* = 17) were refractory. 

Twelve months after therapy initiation, an additional 7.4% (*n* = 7) showed endoscopic improvement, 6.3% (*n* = 6) showed endoscopic remission, and 9.5% (*n* = 9) were refractory. A total of 76.8% of data were not available at this time point. The progression of the individual patients between months 6 and 12 could not be compared because endoscopy data were available at either 6 months or 12 months, but not at both times.

Endoscopic outcomes at the time of the last follow-up showed a response in 20.0% (*n* = 19), remission in 29.5% (*n* = 28), and 24.2% (*n* = 23) were refractory. 26.3% of the data were unknown.

There was no significant difference (*p* = 0.728) (Table S13) comparing patients with CD and those with UC in endoscopic outcomes. It could be shown that patients who received therapy escalation were significantly more likely to be endoscopically refractory (34.4% vs. 6.1%, *p* = 0.017) (Table S14). A total of 19.6% (*n* = 10) of patients with 0–2 prior therapies, 30.0% (*n* = 9) of patients with 3–4 previous treatments, and 33.3% (*n* = 4) of patients with 5 or more prior therapies were endoscopically refractory. Remission was more frequent in patients with 0–2 prior therapies and 5 or more prior therapies than in patients with 3–4 treatments (47.1% and 16.7% vs. 6.7%, *p* ≤ 0.01).

### 3.7. Response of Biomarker

**Figure 1:**
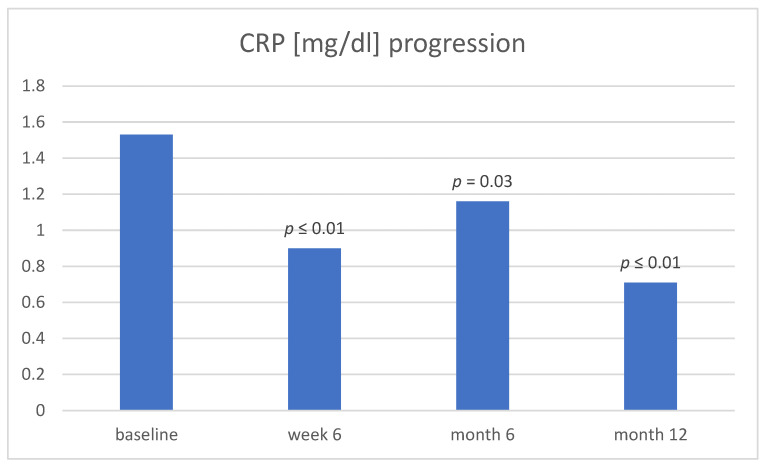
CRP at baseline, week 6, month 6, and month 12. Abbreviation: CRP, c-reactive protein.

Treatment response to therapy was also monitored by biomarker response. From baseline to week 6 there was a significant change in CRP levels alone (1.53 mg/dL ± 2.66 vs. 0.90 mg/dL ± 1.13, *p* < 0.01). CRP levels also decreased significantly from baseline to month 6 (1.16 mg/dL ± 1.94, *p* = 0.03) as well as at month 12 (0.71 mg/dL ± 0.88, *p* ≤ 0.01) (Figure 1).

Patients with UC presented a significantly higher Alb at week 6 (42.56 g/L ± 3.92 vs. 39.95 g/L ± 5.34, *p* = 0.046) (Table S15) as well as lower CRP at month 6 (0.74 mg/dL ± 1.10 vs. 2.13 mg/dL ± 3.20, *p* ≤ 0.01) than patients with CD.

**Figure 2:**
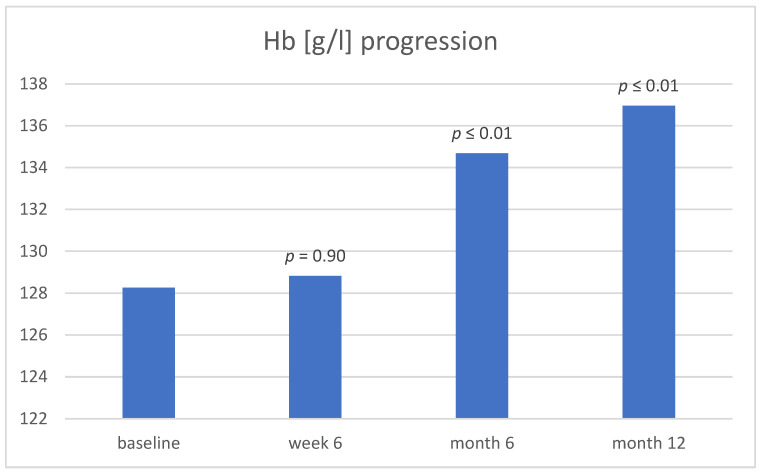
Hb at baseline, week 6, month 6, and month 12. Abbreviation: Hb, hemoglobin.

Hb also showed a significant increase at month 6 (128.26 g/L ± 17.92 vs. 134.68 g/L ± 12.89, *p* ≤ 0.01) and at month 12 (136.95 g/L ± 14.26, *p* ≤ 0.01) (Figure 2).

Baseline Clp and Clp within the first year after VDZ initiation decreased for both groups (CD and UC), but this difference was marginally not significant (488.08 µg/g ± 314.23 vs. 324.83 µg/g ± 330.24, *p* = 0.052).

### 3.8. Steroid-Free Remission

At the start of VDZ treatment, 39 patients (41.1%) of the cohort received steroids concomitantly. Of all 43 patients with clinical remission at the time of the last follow-up, 42 patients were steroid-free (97.7%) (Figure S1) and only 1 patient (2.3%) remained on steroids due to adrenal insufficiency. Of the patients with steroid-free clinical remission, 28 (65.1%) had not been administered corticosteroids during VDZ treatment. During the treatment course, steroid discontinuation was possible in 14 patients (32.6%). A total of 16 (57.1%) (Figure S2) of the 28 patients with endoscopic remission never had steroids during VDZ treatment. In 12 patients (42.9%), discontinuing steroids during treatment was possible.

### 3.9. VDZ Level—Predictor of Clinical Response

At week six, a VDZ trough level was measured in 46 patients (48.4%) (Table 4), and no data were available in 49 patients (51.6%). The average VDZ level in the study was 41.79 µg/mL ± 24.58.

Twenty-five patients with known VDZ levels (54.3%) (Table S16) reported clinical improvement at week 6. The drug level (39.69 µg/mL ± 23.13 vs. 50.8 µg/mL ± 35.01) was not significantly higher with a better outcome at week 6 (*p* = 0.841). It was found that patients who received an additional infusion of VDZ at week 10 previously had a lower VDZ level at week 6 (39.03 µg/mL ± 24.45 vs. 44.55 µg/mL ± 24.93). However, the difference was not significant (*p* = 0.429).

Comparing the group with VDZ level at week 6 with the group without VDZ level, only the clinical outcome at 6 months showed a significantly higher response. It was also significantly less refractory (19.2% vs. 48.6%, 42.3% vs. 13.5%, *p* = 0.033) (Table S17). Between those two groups, there were no significant differences in endoscopic outcomes at 6 months (*p* = 0.343) and clinical and endoscopic outcomes at 12 months (*p* = 0.14; *p* = 0.856).

**Figure 3:**
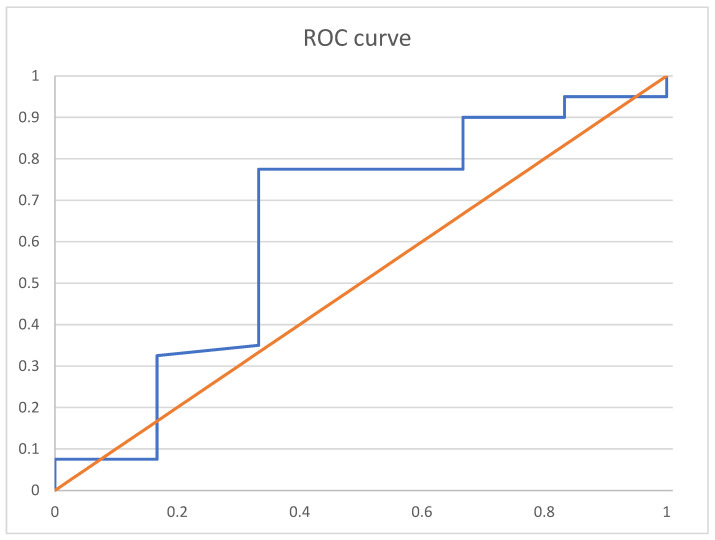
Receiver-operating characteristic (ROC) curve analysis of week 6 vedolizumab trough level and clinical response and remission. ROC curves showing the correlation between VDZ trough level and clinical remission and response: The area under the curve was 0.604. orange: baseline, blue: AUC curve for vedolizumab trough levels and clinical response and remission.

Analyses of the receiver operation characteristics curve for threshold show the following: a VDZ level cut-off of 19 µg/mL (Figure 3) at week 6 predicts clinical remission and clinical response with a sensitivity of 80.0% and a specificity of 33.3% with an area under the receiver operation curve (AUROC) of 0.635.

**Figure 4:**
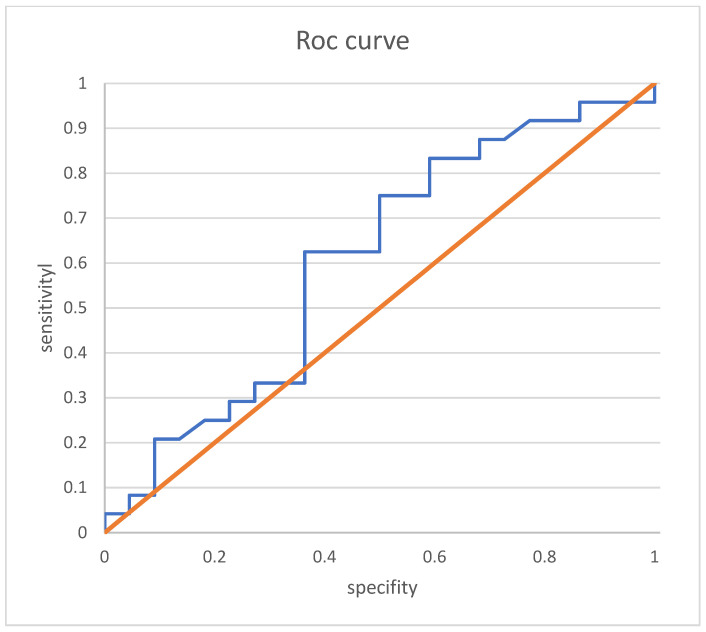
ROC curve analysis of week 6 vedolizumab trough level and clinical remission. ROC curves showing the correlation between VDZ trough level and clinical remission: the area under the curve was 0.635. orange: baseline, blue: AUC curve for vedolizumab trough level and clinical remission.

ROC curve analysis revealed, for clinical remission alone, a VDZ trough level cut-off of 24.5 µg/mL (Figure 4) at week 6 with a sensitivity of 83.3% and a specificity of 41.0%. The AUROC was 0.604.

**Figure 5:**
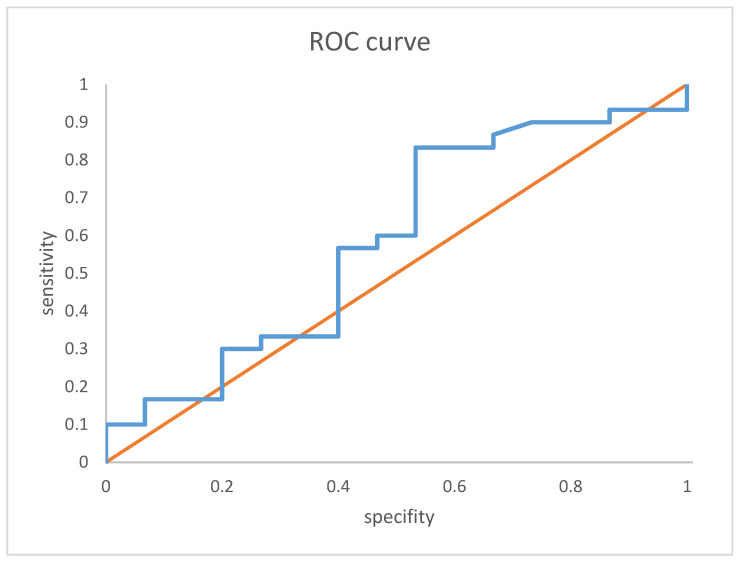
ROC curve analysis of week 6 vedolizumab trough level and endoscopic remission and response. ROC curves showing the correlation between VDZ trough level and endoscopic remission and response: the area under the curve was 0.593. orange: baseline, blue: AUC curve for vedolizumab trough level and endoscopic remission and response.

A VDZ level cut-off of 26.5 µg/mL (Figure 5) was revealed for predicting endoscopic remission and endoscopic response with a sensitivity of 80.0% and a specificity of 47.0%.

**Figure 6:**
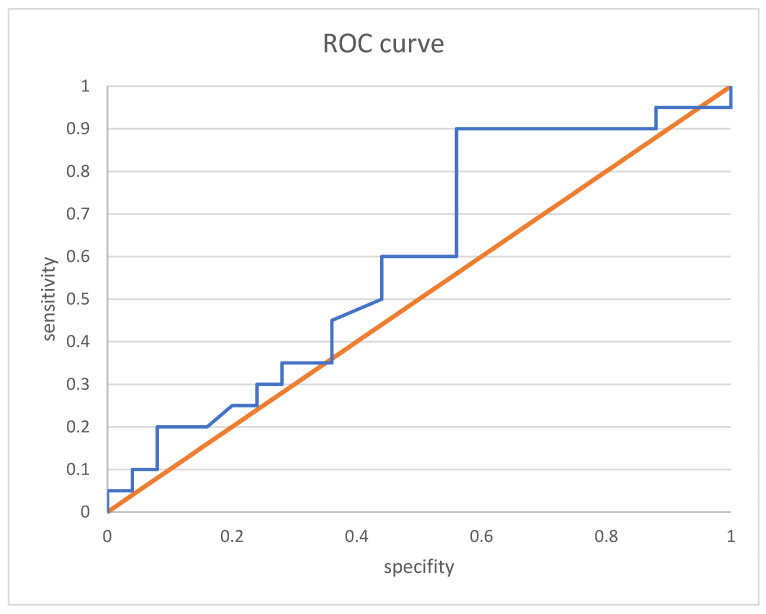
ROC curve analysis of week 6 vedolizumab trough level and endoscopic remission. ROC curves showing the correlation between VDZ trough level and endoscopic remission: The area under the curve was 0.586. orange: baseline, blue: AUC curve for veedolizumab and endoscopic remission.

The AUROC was 0.586. A VDZ trough level cut-off of 30 µg/mL (Figure 6) at week 6 was predictive for endoscopic remission alone with a sensitivity of 80.0% and a specificity of 44.0% with an AUROC of 0.593.

### 3.10. VDZ Level and Long-Term Outcome

Patients who had shown clinical response at month 6 had a mean VDZ level at week 6 of 45.98 µg/mL (*n* = 18) (Table S8), patients in remission had a level of 44.21 µg/mL (*n* = 8), and refractory patients had a level of 42.46 µg/mL (*n* = 5). There was no significant difference in VDZ level regarding clinical response at month 6 (*p* = 0.845).

Patients who had shown endoscopic response at month 6 had a mean VDZ level at week 6 of 40.75 µg/mL (*n* = 8) (Table S12). Patients in remission had a level of 57 µg/mL (*n* = 10), and refractory patients had a level of 34.54 µg/mL (*n* = 9). There was no significant difference in VDZ level regarding endoscopic response at 6 months (*p* = 0.221).

Patients who had shown clinical response at month 12 had a mean VDZ level at week 6 of 55.29 µg/mL (*n* = 7), patients in remission had a level of 39.97 µg/mL (*n* = 9), and refractory patients had a level of 46 µg/mL (*n* = 3). There was no significant difference in VDZ level regarding clinical response at month 12 (*p* = 0.67).

Patients who had shown endoscopic response at month 12 had a mean VDZ level at week 6 of 19.17 µg/mL (*n* = 4), patients in remission had a level of 32.94 µg/mL (*n* = 5), and refractory patients had a level of 31.27 µg/mL (*n* = 6). There was no significant difference in VDZ level regarding endoscopic response at 12 months (*p* = 0.264).

Patients who had shown clinical response at the last follow-up had a mean VDZ level at week 6 of 38. 83 µg/mL (*n* = 16), patients in remission had a level of 45.65 µg/mL (*n* = 24), and refractory patients had a level of 32.58 µg/mL (*n* = 4). There was no significant difference in VDZ level regarding clinical response at the time of the last follow-up (*p* = 0.539).

Patients who had shown endoscopic response at the last follow-up had a mean VDZ level at week 6 of 40.57 µg/mL (*n* = 10), patients in remission had a level of 46.93 µg/mL (*n* = 20), and refractory patients had a level of 35.99 µg/mL (*n* = 11). There was no significant difference in VDZ level regarding endoscopic response at the last follow-up (*p* = 0.708).

### 3.11. Adverse Events (AE)

In the cohort, 71 patients (74.7%) had no relevant AE, 10 (10.5%) patients showed one AE, 7 patients (7.4%) had two AE, and in 7 cases (7.4%), AE were not documented. Skin changes were the most common (23.1%, *n* = 6) (Table 5) AE reported, and respiratory infections were the second most common (19.2%, *n* = 5). GI infections (15.4%, *n* = 4) and headache (15.4%, *n* = 4) were the third most common AE followed by arthralgias (7.7%, *n* = 2) and alopecia (7.7%, *n* = 2). Other AE included fatigue, herpes zoster, and oropharyngeal pain, with 3.8% (*n* = 1) each.

## 4. Discussion

Despite the approval of VDZ in 2014, the role of TDM in VDZ in daily practice remains unclear. Our study at a national expert center for IBD represents an important contribution to the current literature. Few real-world data from outpatients are available in this size.

This study confirmed that VDZ is effective in the long-term in a real-life cohort. Patients with a mean VDZ treatment time of 17.83 months ± 14.56 showed clinical response in 29.5% (*n* = 28) and clinical remission in 45.3% (*n* = 43) at the end of the study. Endoscopic response occurred in 20.0% (*n* = 19) and endoscopic remission in 29.5% (*n* = 28). The sustained beneficial effect of VDZ was also reflected in the biomarker response.

Regarding safety, no new concerns or events occurred in our study [13,14]. Although the comparability of the studies is limited because of differences in study design, definitions, endpoints, and timing of data collection, our VDZ efficacy results align with previous studies.

Our study could not demonstrate a significant relationship between the mean VDZ level at week 6 of 41.79 µg/mL ± 24.58 and both short- and long-term outcomes. However, there was a trend toward numerically higher VDZ levels in patients with clinical and endoscopic response and remission than in nonresponders. Also, comparing the clinical outcome at month 6 between the group with VDZ levels at week 6 with the group without VDZ levels, there was significantly more response and fewer refractory patients in the group with VDZ levels (19.2% vs. 48.6%; 42.3% vs. 13.5%; *p* = 0.033). The value of VDZ monitoring has also been shown in some mainly retrospective studies [15,16,17,18,19,20,21,22,23]. Liefferinckx et al. showed that patients with long-term response had higher levels of VDZ at week 6 compared with nonresponders (33 vs. 42 µg/mL, *p* = 0.02) [21]. A cut-off VDZ level of 28 µg/mL was found to predict long-term response. Also, Yacoub et al. found a difference in VDZ levels at week 6 levels in patients with and without mucosal healing within the first year (26.8 vs. 15.1 µg/mL, *p* = 0.035) [16]. He proposed a cut-off level of 18 µg/mL as a predictor for mucosal healing within the first year. Similarly, we found a cut-off value of 19 µg/mL at week 6 for clinical response and remission and 26.5 µg/mL at week 6 for endoscopic response and remission.

In contrast, there are studies showing evidence to the contrary. Although Williet et al. primarily investigated that a VDZ level of <18.5 µg/mL at week 6 was associated with the need for therapy escalation, there was no difference in VDZ trough levels between responders and nonresponders [24]. Furthermore, in the study by Ungar et al., as in Al-Bawardy et al., there was no difference between clinical or endoscopic responders vs. nonresponders in median VDZ levels determined during maintenance therapy (15.9 vs. 14 µg/mL; 13.7 vs. 16.1 µg/mL) [25,26].

It should be noted that the studies with positive associations are based on real-world data and large register studies. In contrast, the studies that could not exclusively confirm the association are purely real-world data with a relatively small collective. This raises the question of whether an improvement cannot be proven in our case. Therefore, a possible explanation would be the clinical real-world setting in contrast to the setting of registration studies with complete and close-meshed data collection, which does not occur in clinical everyday life for various reasons. Furthermore, it should be noted that in clinical practice, the regular VDZ level determination does not represent an established standard of therapy implementation.

It was also noticed that patient cohorts with positive associations between VDZ levels and outcome response rates were similar to those without or with only weak associations. This raises the question of whether VDZ level has a relevant influence on the subsequent treatment response because a comparably good response should not be observed in our collective or in studies with negative associations. It also casts doubt on the need to achieve a minimum range of VDZ in terms of therapeutic response. Rosario suggested that even low levels of VDZ (1 µg/mL) represented a sufficient amount of VDZ to achieve near-complete saturation of the α4β7 receptor [27]. The current state of research suggests that VDZ interferes with the mucosal innate immune system [28]. These aspects further support that serum levels of VDZ are thus not necessarily subject to a clear dose–response relationship, which, on the one hand, questions the usefulness of drug monitoring with VDZ and, on the other hand, whether it is also likely to achieve clinical significance in terms of cost.

Another rationale that argues against the need for a level-controlled therapy application is the ENTERPRET trial. In this recently published study, patients with UC, who did not show primary response after 5 weeks of treatment with VDZ, were assigned to a VDZ standard dose vs. optimized dose by level. At week 30, there was no relevant difference in MH between the standard-dose arm and dose-optimization arm (18.9% vs. 14.5%), and the results were also similar in clinical outcome [29]. Thus, despite the clinical consequence of increasing the dose to lower VDZ levels, there were no improved outcomes.

It would be interesting to have data comparing a dose reduction with the current standard dose to support the previously mentioned mechanisms (saturation of the α4β7 receptor, effects on the innate immune response) with a further given treatment efficacy.

That outcome is not affected by lower VDZ levels was also shown by several studies from 2020 and 2021: Vermeire et al. examined the efficacy of a cohort with VDZ interval reduction from four to eight weeks. Although the average VDZ level decreased from 43.6 µg/mL to 10.4 µg/mL at week 56 91.0% of CD patients and 92.0% of UC patients remained on eight-week VDZ therapy for at least 56 weeks [30]. However, the disease was very well-controlled in this collective, with some patients receiving VDZ for over six years. In contrast, Outtier et al. demonstrated that dose escalation from eight to four weeks in patients with loss of response resulted in regaining response in almost half of patients. Nevertheless, baseline VDZ trough level did not predict response to dose escalation, implying that TDM is not indicated in VDZ-treated patients [31]. Also, Ungar et al. demonstrated that lower VDZ levels before dose optimization did not predict success, thus arguing against a pharmacokinetic basis for insufficient response to VDZ [32].

The question arises whether the present discrepant results may be an expression of the underlying biology of the disease and, therefore, that VDZ levels may be a surrogate marker of disease activity rather than VDZ efficacy in individuals [17].

In this context, parameters are urgently needed to correlate the biological efficacy of VDZ with clinical outcome since the measured serum levels, as shown by the discrepant published data, do not fulfill this criterion beyond doubt. If the efficacy of VDZ were correlated with the level, a simple increase in the applied dose should lead to better responses. But this was negated by the ENTERPRET trial.

Although already known predictive factors such as anti-TNF naive, female sex, and higher albumin could be confirmed in this study, there is a need for a clear parameter of the biological efficacy of VDZ [15,27].

The limitations of our study are the retrospective, monocentric character of the data collection. Furthermore, not all patients treated with VDZ underwent VDZ level measurement or underwent standardized analysis, e.g., with a scoring system due to the retrospective character of the study. A strength of our study is the clinical real-life aspect and the large number of patients due to the monocentric structure.

In conclusion, this study demonstrates efficacy and safety for the VDZ-treated population. However, monitoring VDZ efficacy by serum level is not a clinically relevant and useful option for therapy management. Nevertheless, we have identified possible cut-off values as therapeutic targets and demonstrated that numerical data show that higher levels result in better outcomes. However, an explicit parameter of biological efficacy for VDZ is needed and should be investigated in further clinical trials.

## Figures and Tables

**Table 1:** IBD-associated complications prior VDZ.

	*n*	%
abscess	11	14.5
fistula	16	21.1
fissure	7	9.2
stenosis	23	30.3
intraepithelial dysplasia	5	6.6
carcinoma	4	5.3
ileus/subileus	5	6.6
malabsorption syndrome	2	2.6
toxic megacolon	1	1.3
CMV colitis	2	2.6
total	76	100

Abbreviation: CMV, cytomegalovirus; IBD, inflammatory bowel disease; VDZ, vedolizumab.

**Table 2:** Biomarkers at baseline.

Diagnosis		bl Hb (g/L)	bl CRP (mg/dL)	bl Clp (µg/g)	bl Fe (ng/mL)	bl Alb (g/L)
CD	average	132.91	1.77	495.05	274.74	41.83
	sd	13.77	1.96	293.13	399.62	5.94
UC	average	126.67	1.56	473.5	108.02	42.19
	sd	19.4	2.99	328.16	166.03	5.25
*p*-value		0.524	0.177	0.618	0.036	0.812

Abbreviation: Alb, albumin; bl, baseline; CD, Crohn´s disease; CI, colitis indeterminata; Clp, calprotectin; Crp, c-reactive protein; Fe, ferritin; Hb, hemoglobin; UC, ulcerative colitis; sd, standard deviation.

**Table 3:** Frequency of used agents and therapy regimes before VDZ.

Agents Used		*n*	%
	5-ASA	87	30.2
	immunosuppressants	93	32.3
	tumor necrosis factor antagonists	92	31.9
	januskinase-inhibitors	2	0.7
	interleukin 12/23 antagonists	9	3.1
	others	5	1.7
	total	288	100
Therapy Regime	monotherapy	222	87.4
	dual combination therapy	30	11.8
	tripe combination therapy	2	0.8
	total	254	100

**Table 4:** VDZ level at week 6 (ug/mL).

Valid	46
average	41.79
median	38.5
sd	24.577
range	96
min	5
max	101

Abbreviation: max, maximum; min, minimum; sd, standard deviation; VDZ, vedolizumab.

**Table 5:** Adverse events.

	*n*	%
fatigue	1	3.8
gastrointestinal infections	4	15.4
alopecia	2	7.7
skin changes	6	23.1
headache	4	15.4
arthralgia	2	7.7
respiratory infections	5	19.2
herpes zoster	1	3.8
oropharyngeal pain	1	3.8
total	26	100

## Data Availability

All data generated or analyzed during this study are included in this article and its supplementary materials. Further inquiries can be directed to the corresponding author.

## Supplementary Materials

The following supporting information can be downloaded at: https://www.mdpi.com/article/10.3390/jcm13010140/s1. Figure S1: Steroid-free clinical remission at time of last follow-up; Figure S2: Steroid-free endoscopic remission at time of last follow-up; Table S1: Patient demographics and the characteristics of treatment with VDZ; Table S2: Patient characteristics at baseline; Table S3: Extraintestinal manifestations; Table S4: Comparison of baseline characteristics between CD, UC, and CI; Table S5: Biomarkers at baseline; Table S6: Spearman’s correlation between prior therapies, remission duration, VDZ level at week 6, and disease duration until VDZ; Table S7: Comparison of prior therapies between CD and UC; Table S8: Clinical outcome with VDZ level week 6 at different timepoints; Table S9: Sex-related clinical outcome at last follow-up; Table S10: Comparison of clinical outcome at last follow up in CD, UC, and CI; Table S11: Association of therapy escalation and clinical outcome at last follow up; Table S12: Endoscopic outcome with VDZ level week 6 at different timepoints; Table S13: Comparison of endoscopic outcome at last follow-up in CD, UC, and CI; Table S14: Association of therapy escalation and endoscopic outcome at last follow up; Table S15: Biomarker response at week 6, month 6, and month 12; Table S16: Clinical improvement at week 6 and additional dose receivers in week 10 with VDZ trough level at week 6; Table S17: Clinical and endoscopic outcome in patients with and without VDZ level week 6.

## Abbreviations

AE	Adverse event
Alb	Albumin
BMI	Body mass index
Clp	Calprotectin
CI	Colitis indeterminate
CRC	Colorectal cancer
CD	Crohn’s disease
CRP	C-reactive protein
EMA	European Medicines Agency
Fe	Ferritin
GI	Gastrointestinal
Hb	Hemoglobin
IBD	Inflammatory bowel disease
i.v.	Intravenous
JAK	Janus kinase
MRI	Magnetic resonance imaging
MH	Mucosal healing
PSC	Primary sclerosing cholangitis
ROC	Receiver-operating characteristic
rr	Reference range
S1P	Sphingosine-1-phosphate receptor modulators
s.c.	Subcutaneous
TDM	Therapeutic drug monitoring
TNF	Tumor necrosis factor
UC	Ulcerative colitis
VDZ	Vedolizumab
