# Supplementary material for: Efficacy and Safety of Vedolizumab in Patients with Inflammatory Bowel Disease in Association with Vedolizumab Drug Levels
Source: J Clin Med. 2023 Dec 27;13(1):140. doi: 10.3390/jcm13010140 (PMC10779856; doi:10.3390/jcm13010140)
Supplement: Supplementary file 1 [file jcm-13-00140-s001.zip › Tabel S3.pdf]

Table S3. Extraintestinal manifestations: Twenty-three patients showed one extraintestinal manifestation; nine showed two.

	**n**	**%**
erythrema nodosum	5	12.2
pyoderma gangrenosum	2	4.9
joint involvement	20	48.8
eye involvement	8	19.5
PSC	6	14.6
**total**	**41**	**100**

Abbreviation: PSC, primary sclerosing colitis
