# Supplementary material for: Efficacy and Safety of Vedolizumab in Patients with Inflammatory Bowel Disease in Association with Vedolizumab Drug Levels
Source: J Clin Med. 2023 Dec 27;13(1):140. doi: 10.3390/jcm13010140 (PMC10779856; doi:10.3390/jcm13010140)
Supplement: Supplementary file 1 [file jcm-13-00140-s001.zip › Table S1.pdf]

Table S1. Patient demographics and characteristics of treatment with VDZ.

		n	%	average	sd	p-value
female		47	49.5			
male		48	50.5			
CD		23	24.2			
UC		65	68.4			
CI		7	7.4			
age at diagnosis				30.87	14.68	
age at start of VDZ therapy				41	15.28	
disease duration at start of VDZ [years]				10.5	9	
duration of VDZ therapy [months]				17.8	14.6	
application i.v.		82	86.3			
application s.c.		13	13.7			
additional infusion at week 10		35	36.8			
	CD	12	34.3			
	UC	22	62.9			
	CI	1	2.9			0.217
escalation of therapy		62	65.3			
re-induction		2	2.1			
under VDZ at last follow up		47	49.5			
discontinuation of VDZ at last follow up		48	50.5			
	primary nonresponder	19	39.6			
	secondary nonresponder	21	43.8			
	patient´s decision	8	16.7			

Abbreviation**:** CD, Crohn´s disease; CI, colitis indeterminata; i.v., intravenous; s.c., subcutaneous; sd, standard deviation; UC, ulcerative colitis; VDZ, vedolizumab
