# Supplementary material for: Efficacy and Safety of Vedolizumab in Patients with Inflammatory Bowel Disease in Association with Vedolizumab Drug Levels
Source: J Clin Med. 2023 Dec 27;13(1):140. doi: 10.3390/jcm13010140 (PMC10779856; doi:10.3390/jcm13010140)
Supplement: Supplementary file 1 [file jcm-13-00140-s001.zip › Table S10.pdf]

Table S10. Comparison of clinical outcome at last follow up in CD, UC and CI.

	**CD**		**UC**		**CI**		**total**		**p-value**
	n	%	n	%	n	%	n	%	
response	5	23.80	21	33.90	2	28.60	28	31.10	
remission	8	38.10	32	51.60	4	57.10	44	48.90	
refractory	6	28.60	8	12.90	1	14.30	15	16.70	
stable	2	9.50	1	1.60	0	0	3	3.30	0.331
total	21	100	62	100	7	100	90	100	

Abbreviation: CD, Crohn´s disease; CI, colitis indeterminata; UC ulcerative colitis
