# Supplementary material for: Efficacy and Safety of Vedolizumab in Patients with Inflammatory Bowel Disease in Association with Vedolizumab Drug Levels
Source: J Clin Med. 2023 Dec 27;13(1):140. doi: 10.3390/jcm13010140 (PMC10779856; doi:10.3390/jcm13010140)
Supplement: Supplementary file 1 [file jcm-13-00140-s001.zip › Table S11.pdf]

Table S11. Association of therapy escalation and clinical outcome at last follow up.

		**therapy escalation**		**total**	**p-value**
		no	yes		
response	n	9	19	28	
	% of therapy escalation	27.30	30.60	29.50	
remission	n	20	23	43	
	% of therapy escalation	60.60	37.10	45.30	
refractory	n	3	13	16	
	% of therapy escalation	9.10	21.00	16.80	
unknown	n	1	4	5	
	% of therapy escalation	3.00	6.50	5.30	
stable	n	0	3	3	
	% of therapy escalation	0.00	4.80	3.20	
**total**	**n**	**33**	**62**	**95**	**0.159**
	**% of therapy escalation**	**100**	**100**	**100**	
