# Supplementary material for: Efficacy and Safety of Vedolizumab in Patients with Inflammatory Bowel Disease in Association with Vedolizumab Drug Levels
Source: J Clin Med. 2023 Dec 27;13(1):140. doi: 10.3390/jcm13010140 (PMC10779856; doi:10.3390/jcm13010140)
Supplement: Supplementary file 1 [file jcm-13-00140-s001.zip › Table S12.pdf]

Table S12. Endoscopic outcome with VDZ level week 6 at different timepoints.

		**response**	**remission**	**refractory**	**unknown**	**p-value**
month 6	n	15	12	17	51	0.221
	%	15.8	12.6	17.9	53.7	
	VDZ level week 6 [ug/ml]	40.75 ± 25.76	57 ± 26.30	34.54 ± 27.15	-	
	n	8	10	9	-	
month 12	n	7	6	9	73	0.264
	%	7.4	6.3	9.5	76.8	
	VDZ level week 6 [ug/ml]	19.17 ± 12.76	32.94 ± 19.55	31.27 ± 20.07	-	
	n	4	5	6	-	
last follow-up	n	19	28	23	25	0.708
	%	20	29.5	24.2	26.3	
	VDZ level week 6 [ug/ml]	40.57 ± 26.16	46.93 ± 24.72	35.99 ± 24.79	-	
	n	10	20	11	-	

Abbreviation**:** VDZ, vedolizumab
