# Supplementary material for: Efficacy and Safety of Vedolizumab in Patients with Inflammatory Bowel Disease in Association with Vedolizumab Drug Levels
Source: J Clin Med. 2023 Dec 27;13(1):140. doi: 10.3390/jcm13010140 (PMC10779856; doi:10.3390/jcm13010140)
Supplement: Supplementary file 1 [file jcm-13-00140-s001.zip › Table S13.pdf]

Table S13. Comparison of endoscopic outcome at last follow up in CD, UC and CI.

	**CD**		**UC**		**CI**		**total**		**p-value**
	n	%	n	%	n	%	n	%	
response	3	17.60	14	28	2	40	19	26.40	
remission	6	35.30	20	40	2	40	28	38.90	
refractory	8	47.10	16	32	1	20	25	34.70	0.728
total	17	100	50	100	5	100	72	100	

Abbreviation: CD, Crohn´s disease; CI, colitis indeterminata; UC ulcerative colitis
