# Supplementary material for: Efficacy and Safety of Vedolizumab in Patients with Inflammatory Bowel Disease in Association with Vedolizumab Drug Levels
Source: J Clin Med. 2023 Dec 27;13(1):140. doi: 10.3390/jcm13010140 (PMC10779856; doi:10.3390/jcm13010140)
Supplement: Supplementary file 1 [file jcm-13-00140-s001.zip › Table S14.pdf]

Table S14. Association of therapy escalation and endoscopic outcome at last follow up.

		**therapy escalation**		**total**	**p-value**
		no	yes		
response	n	7	12	19	
	% of therapy escalation	21.20	19.70	20.20	
remission	n	12	16	28	
	% of therapy escalation	36.40	26.20	29.80	
refractory	n	2	21	23	
	% of therapy escalation	6.10	34.40	24.50	0.017
unkown	n	12	12	24	
	% of therapy escalation	36.40	19.70	25.50	
total	n	33	61	94	
	% of therapy escalation	100	100	100	
