# Supplementary material for: Efficacy and Safety of Vedolizumab in Patients with Inflammatory Bowel Disease in Association with Vedolizumab Drug Levels
Source: J Clin Med. 2023 Dec 27;13(1):140. doi: 10.3390/jcm13010140 (PMC10779856; doi:10.3390/jcm13010140)
Supplement: Supplementary file 1 [file jcm-13-00140-s001.zip › Table S15.pdf]

Table S15.1. Biomarker response at week 6, month 6 and month 12.

diagnosis	CD		UC		CI		total		p-value
	average	sd	average	sd	average	sd	average	sd	
VDZ level at week 6 [ug/ml]	50.87	30.60	41.49	23.30	21.10	10.41	41.79	24.58	0.20
hemoglobin [g/l] at week 6	129.04	11.06	128.41	14.02	132.17	15.08	128.82	13.28	0.88
CRP [mg/dl] at week 6	1.03	1.07	0.91	1.19	0.36	0.43	0.90	1.13	0.13
calprotectin [µg/g] within one year after VDZ	419.13	349.41	299.41	328.78	140.25	132.58	324.83	330.24	0.38
ferritin [ng/ml] at week 6	164.57	213.79	133.93	186.95	233.40	236.80	147.51	195.87	0.17
albumin [g/l] at week 6	39.95	5.34	42.56	3.92	43.68	4.39	41.97	4.46	0.05
CRP at month 6	2.13	3.20	0.74	1.10	2.45	0.49	1.16	1.94	0.01
hemoglobin at month 6	133.42	17.10	134.91	11.55	137.50	8.70	134.68	12.89	0.95
CRP at months 12	1.03	1.15	0.62	0.76	0.21	0.10	0.71	0.88	0.39
hemoglobin at month 12	131.64	17.25	138.44	12.95	141.33	15.28	136.95	14.26	0.40

Abbreviation: CD, Crohn´s disease; CI, colitis indeterminata; CRP, C-reactive protein; sd, standard deviation; UC, ulcerative colitis; VDZ, Vedolizumab

Table S15~~2~~.2. Pairwise comparison of diagnosis (for Albumin).

Sample 1-Sample 2	test statistics	standard error	standard test statistics	p-value	adjusted p-value
CD-UC	-13,845	6,006	-2,305	0,021	0,063
CD-CI	-19,925	11,473	-1,737	0,082	0,247
UC-CI	-6,08	10,726	-0,567	0,571	1

Abbreviation: CD, Crohn´s disease; CI, Colitis indeterminata; UC, ulcerative colitis

Each row tests the null hypothesis that the distributions in sample 1 and sample 2 are the same.

Asymptotic significances (two-sided tests) are displayed. The significance level is .050.

Adjusted significance values are adjusted by the Bonferroni correction for multiple tests.

Table S15~~2~~.3. Pairwise comparison of diagnosis (for C-reactive protein).

**Sample 1-Sample 2**	**test statistics**	**standard error**	**standard test statistics**	**p-value**	**adjusted p-value**
UC-CD	12,044	5,736	2,1	0,036	0,107
UC-CI	-30,794	12,43	-2,477	0,013	0,04
CD-CI	-18,75	13,048	-1,437	0,151	0,452

Abbreviation: CD, Crohn´s disease; CI, Colitis indeterminata; UC, ulcerative colitis

Each row tests the null hypothesis that the distributions in sample 1 and sample 2 are the same.

Asymptotic significances (two-sided tests) are displayed. The significance level is .050.

Adjusted significance values are adjusted by the Bonferroni correction for multiple tests.
