# Supplementary material for: Efficacy and Safety of Vedolizumab in Patients with Inflammatory Bowel Disease in Association with Vedolizumab Drug Levels
Source: J Clin Med. 2023 Dec 27;13(1):140. doi: 10.3390/jcm13010140 (PMC10779856; doi:10.3390/jcm13010140)
Supplement: Supplementary file 1 [file jcm-13-00140-s001.zip › Table S16.pdf]

Table S16. Clinical improvement at week 6 and additional dose receivers in week 10 with VDZ trough level at week 6.

	**VDZ level week 6 [µg/ml]**			**p-value**
**clinical improvement at week 6**	average	n	sd	
unknown	42.25	16	24.38	0.841
no improvement	50.8	5	35.01	
improvement	39.69	25	23.132	
total	41.79	46	24.577	
**additional dose in Week 10**				
yes	39.03	23	24.454	
no	44.55	23	24.93	
total	41.79	46	24.577	0.429

Abbreviation: sd, standard deviation; VDZ, vedolizumab
