# Supplementary material for: Efficacy and Safety of Vedolizumab in Patients with Inflammatory Bowel Disease in Association with Vedolizumab Drug Levels
Source: J Clin Med. 2023 Dec 27;13(1):140. doi: 10.3390/jcm13010140 (PMC10779856; doi:10.3390/jcm13010140)
Supplement: Supplementary file 1 [file jcm-13-00140-s001.zip › Table S17.pdf]

Table S17. 4. Clinical and endoscopic outcome in patients with and without VDZ level week 6.

		**VDZ level week 6**				**total**		**p-value**
		no		yes				
		n	%	n	%	n	%	
clinical outcome month 6	response	5	19.2	18	48.6	23	36.5	0.03
	remission	5	19.2	8	21.6	13	20.6	
	refractoy	11	42.3	5	13.5	16	25.4	
	unknown	5	19.2	6	16.2	11	17.5	
total		26	100	37	100	63	100	
endoscopic outcome month 6	response	7	33.3	8	24.2	15	27.8	0.34
	remission	2	9.5	10	30.3	12	22.2	
	refractoy	8	38.1	9	27.3	17	31.5	
	unknown	4	19.0	6	18.2	10	18.5	
total		21	100	33	100	54	100	
clinical outcome month 12	response	1	5.3	7	23.3	8	16.3	0.14
	remission	4	21.1	9	30.0	13	26.5	
	refractoy	6	31.6	3	10.0	9	18.4	
	unknown	8	42.1	11	36.7	19	38.8	
total		19	100	30	100	49	100	
endoscopic outcome month 12	response	3	20.0	4	14.8	7	16.7	0.86
	remission	1	6.7	5	18.5	6	14.3	
	refractoy	3	20.0	6	22.2	9	21.4	
	unknown	8	53.3	12	44.4	20	47.6	
total		15	100	27	100	42	100	

Abbreviation: VDZ, vedolizumab

It ist noted that p-values were calculated with the Fisher exact test
