# Supplementary material for: Efficacy and Safety of Vedolizumab in Patients with Inflammatory Bowel Disease in Association with Vedolizumab Drug Levels
Source: J Clin Med. 2023 Dec 27;13(1):140. doi: 10.3390/jcm13010140 (PMC10779856; doi:10.3390/jcm13010140)
Supplement: Supplementary file 1 [file jcm-13-00140-s001.zip › Table S2.pdf]

**Table S2.** Patients characteristics at baseline.

| | **n** | **%** | **average** | **sd** |
|---|---|---|---|---|
| BMI [kg/m²] | | | 23.6 | 4.41 |
| moderate or high symptom burden at baseline | 67 | 70.5 | | |
| presence of at least one extraintestinal manifestation | 32 | 33.7 | | |
| IBD complications at baseline | 43 | 45.3 | | |
| prior bowel surgery | 25 | 26.3 | | |
| UC | 8 | 32 | | |
| CD | 17 | 68 | | |

Abbreviation: BMI, body mass index; CD, Crohn's disease; IBD, inflammatory bowel disease; sd, standard deviation; UC, ulcerative colitis
