# Supplementary material for: Efficacy and Safety of Vedolizumab in Patients with Inflammatory Bowel Disease in Association with Vedolizumab Drug Levels
Source: J Clin Med. 2023 Dec 27;13(1):140. doi: 10.3390/jcm13010140 (PMC10779856; doi:10.3390/jcm13010140)
Supplement: Supplementary file 1 [file jcm-13-00140-s001.zip › Table S4.pdf]

Table S4. 1.Comparison of baseline characteristics between CD, UC and CI.

**diagnosis**	**total**		**CD**		**UC**		**CI**		**p-value**
	average	sd	average	sd	average	sd	average	sd	
age	44.80	15.34	42.52	13.91	44.66	15.81	53.57	14.20	0.23
age at diagnosis	30.87	14.69	26.00	14.59	32.34	13.75	33.29	21.49	0.09
disease duration	13.08	9.24	15.53	7.04	11.48	9.30	19.87	11.31	0.01
BMI	23.61	4.44	22.44	3.71	24.31	4.64	22.47	4.73	0.32

Abbreviation: BMI, body mass index; CD, Crohn´s disease; CI, Colitis indeterminata; sd, standard deviation; UC, ulcerative colitis

Table S4. 2. Pairwise comparison of diagnosis.

sample 1-sample 2	p-value	adjusted p-value
UC-CD	0,008	0,023
UC-CI	0,024	0,073
CD-CI	0,562	1

Abbreviation: CD, Crohn´s disease; CI, Colitis indeterminata; UC, ulcerative colitis

Each row tests the null hypothesis that the distributions in sample 1 and sample 2 are the same.

Asymptotic significances (two-sided tests) are displayed. The significance level is .050.

Adjusted significance values are adjusted by the Bonferroni correction for multiple tests.
