# Supplementary material for: Efficacy and Safety of Vedolizumab in Patients with Inflammatory Bowel Disease in Association with Vedolizumab Drug Levels
Source: J Clin Med. 2023 Dec 27;13(1):140. doi: 10.3390/jcm13010140 (PMC10779856; doi:10.3390/jcm13010140)
Supplement: Supplementary file 1 [file jcm-13-00140-s001.zip › Table S5.pdf]

Table S5.1. Biomarkers at baseline.

diagnosis		bl Hb [g/l]	bl CRP [mg/dl]	bl Clp [µg/g]	bl Fe [ng/ml]	bl Alb [g/l]
CD	average	132.91	1.77	495.05	274.74	41.83
	sd	13.77	1.96	293.13	399.62	5.94
UC	average	126.67	1.56	473.50	108.02	42.19
	sd	19.40	2.99	328.16	166.03	5.25
CI	average	125.33	0.64	662.33	154.00	43.07
	sd	13.56	0.78	238.45	133.59	3.85
p-value		0.524	0.177	0.618	0.036	0.812

Abbreviations: Alb, albumin; bl, baseline; CD, Crohn´s disease; CI, colitis indeterminata; Clp, Calprotectin; CRP, C-reactive protein; Fe, Ferritin; Hb, Hemoglobin; UC, ulcerative colitis; sd, standard deviation

Table S5.2. Pairwise comparison of diagnosis (for Fe).

sample 1-sample 2	p-value	adjusted p-value
UC-CD	0,031	0,092
UC-CI	0,082	0,245
CD-CI	0,642	1

Abbreviation: CD, Crohn´s disease; CI, Colitis indeterminata; UC, ulcerative colitis

Each row tests the null hypothesis that the distributions in sample 1 and sample 2 are the same.
Asymptotic significances (two-sided tests) are displayed. The significance level is .050.
Adjusted significance values are adjusted by the Bonferroni correction for multiple tests.
