# Supplementary material for: Efficacy and Safety of Vedolizumab in Patients with Inflammatory Bowel Disease in Association with Vedolizumab Drug Levels
Source: J Clin Med. 2023 Dec 27;13(1):140. doi: 10.3390/jcm13010140 (PMC10779856; doi:10.3390/jcm13010140)
Supplement: Supplementary file 1 [file jcm-13-00140-s001.zip › Table S6.pdf]

Table S6. Spearman correlation between prior therapies, remission duration, VDZ level at week 6 and disease duration until VDZ.

			remission duration	VDZ level at week 6 [µg/ml]	disease duration until VDZ [months]
spearman-rho	number of prior therapies	correlation coefficient	-0.289	-0.033	0.569
		p-value	0.007	0.825	0.00
		n	86	45	94

Abbreviation: VDZ, vedolizumab
