# Supplementary material for: Efficacy and Safety of Vedolizumab in Patients with Inflammatory Bowel Disease in Association with Vedolizumab Drug Levels
Source: J Clin Med. 2023 Dec 27;13(1):140. doi: 10.3390/jcm13010140 (PMC10779856; doi:10.3390/jcm13010140)
Supplement: Supplementary file 1 [file jcm-13-00140-s001.zip › Table S7.pdf]

Table S7.1. Comparison of prior therapies between CD and UC.

	**average**	**sd**	**p-value**
CD	4	1.574	
UC	2.26	1.524	
CI	2.71	1.704	
total	2.7	1.696	<0.01

Abbreviation: CI, colitis indeterminata; CD, Crohn´s disease; sd, standard deviation; UC, ulcerative colitis

Table S7.2. Pairwise comparison of diagnosis.

**sample 1-sample 2**	**test statistics**	**standard error**	**standard test statistics**	**p-value**	**adjusted p-value**
UC-CI	-7,923	10,607	-0,747	0,455	1
UC-CD	29,196	6,577	4,439	<0.01	<0.01
CI-CD	21,273	11,571	1,838	0,066	0,198

Abbreviation: CD, Crohn´s disease; CI, Colitis indeterminata; UC, ulcerative colitis

Each row tests the null hypothesis that the distributions in sample 1 and sample 2 are the same.

Asymptotic significances (two-sided tests) are displayed. The significance level is .050.

Adjusted significance values are adjusted by the Bonferroni correction for multiple tests.
