# Supplementary material for: Efficacy and Safety of Vedolizumab in Patients with Inflammatory Bowel Disease in Association with Vedolizumab Drug Levels
Source: J Clin Med. 2023 Dec 27;13(1):140. doi: 10.3390/jcm13010140 (PMC10779856; doi:10.3390/jcm13010140)
Supplement: Supplementary file 1 [file jcm-13-00140-s001.zip › Table S8.pdf]

Table S8. Clinical outcome with VDZ level week 6 at different timepoints.

		response	remission	refractory	stable	unknown	p-value
month 6	n	23	13	16	-	43	0.845
	%	24.2	13.7	16.8	-	45.3	
	VDZ level week 6 [ug/ml]	45.98 ± 26.0	44.21 ± 30.56	42.46 ± 28.47	-	-	
	n	18	8	5	-	-	
month 12	n	8	13	9	-	65	0.67
	%	8.4	13.7	9.5	-	68.4	
	VDZ level week 6 [ug/ml]	55.29 ± 23.62	39.97 ± 26.23	46 ± 26.46	-	-	
	n	7	9	3	-	-	
last follow-up	n	28	43	16	3	5	0.539
	%	29.5	45.3	16.8	3.2	5.3	
	VDZ level week 6 [ug/ml]	38.83 ± 24.19	45.65 ± 24.25	32.58 ± 34.85	24 ± 0	-	
	n	16	24	4	1	-	

Abbreviation**:** VDZ, vedolizumab
