# Supplementary material for: Efficacy and Safety of Vedolizumab in Patients with Inflammatory Bowel Disease in Association with Vedolizumab Drug Levels
Source: J Clin Med. 2023 Dec 27;13(1):140. doi: 10.3390/jcm13010140 (PMC10779856; doi:10.3390/jcm13010140)
Supplement: Supplementary file 1 [file jcm-13-00140-s001.zip › Table S9.pdf]

Table S9. Sex-related clinical outcome at last follow up.

		female		male		total		p-value
		**n**	**%**	**n**	**%**	**n**	**%**	
clinical outcome at last follow up	response	9	20.50	19	41.3	28	31.10	
	remission	27	61.40	17	37	44	48.90	
	refractory	7	15.90	8	17	15	16.70	
	stable	1	2.30	2	4.30	3	3.30	0.098
total		44	100.00	46	1.00	90	100.00	
