# Supplementary figures and images for: Efficacy and Safety of Vedolizumab in Patients with Inflammatory Bowel Disease in Association with Vedolizumab Drug Levels
Source: J Clin Med. 2023 Dec 27;13(1):140. doi: 10.3390/jcm13010140 (PMC10779856; doi:10.3390/jcm13010140)

**Figure S1.** Steroidfree clinical remission at time of last follow-up.

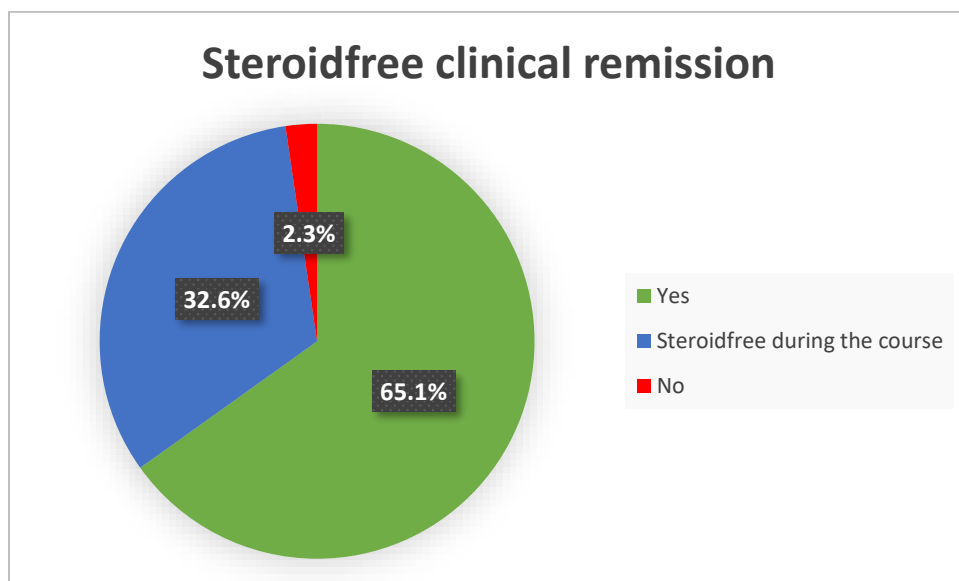

Supplement: Supplementary file 1 [file jcm-13-00140-s001.zip › Figure S1.pdf]

**Figure S2.** Steroidfree endoscopic remission at time of last follow-up.

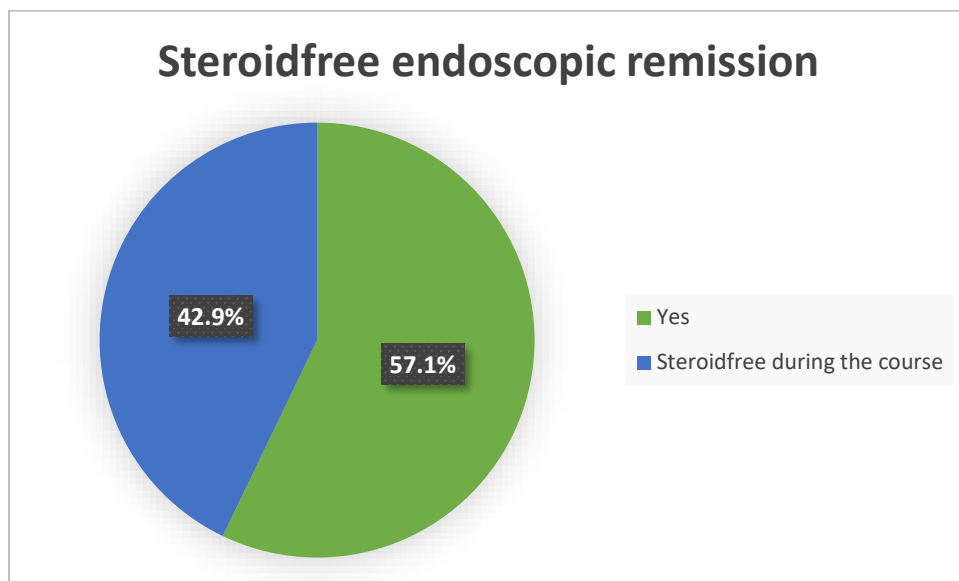

Supplement: Supplementary file 1 [file jcm-13-00140-s001.zip › Figure S2.pdf]
